# Interlayer Electric Multipole Hall Effect in Twisted Multilayers

**DOI:** 10.1021/acs.nanolett.6c01563

**Published:** 2026-07-25

**Authors:** Chengxin Xiao, Cong Xiao, Dawei Zhai, Wang Yao

**Affiliations:** † New Cornerstone Science Laboratory, Department of Physics, 25809 The University of Hong Kong, Hong Kong 999077, China; ‡ School of Electrical and Information Engineering, Zhengzhou University, Zhengzhou, Henan 450001, China; ¶ HK Institute of Quantum Science & Technology, 25809 The University of Hong Kong, Hong Kong 999077, China; § Interdisciplinary Center for Theoretical Physics and Information Sciences (ICTPIS), Fudan University, Shanghai 200433, China; ∥ State Key Laboratory of Surface Physics, Fudan University, Shanghai 200433, China; ⊥ State Key Laboratory of Optical Quantum Materials, 25809 The University of Hong Kong, Hong Kong 999077, China

**Keywords:** layer Hall effect, moiré superlattice, twisted trilayer graphene, interlayer electric quadrupole, in-plane magnetic quadrupole

## Abstract

Electrons in layered van der Waals materials possess a layer pseudospin characterizing their wave function distribution among layers. In twisted structures, this pseudospin forms nontrivial textures, leading to intriguing phenomena such as the layer Hall effect (LHE), where distinct layer Hall currents flow, despite the presence of time-reversal symmetry. In chiral bilayers, the LHE manifests as an interlayer electric dipole Hall effect with Hall counterflows and a concomitant in-plane magnetic dipole. The multilayer hosts richer layer-dependent Hall currents, generating interlayer electric multipole Hall effects and in-plane magnetic multipoles. We start by exploring the interlayer electric quadrupole Hall effect in mirror-symmetric twisted trilayers. At small twist angles, interlayer translation efficiently tunes layer Hall current magnitudes. At large angles and low doping, the currents can be well-accounted for by adding the contributions from the two individual twisted interfaces. This decomposition allows us to obtain layer-resolved Hall currents in large-angle twisted multilayers even without well-defined periodicity.

## Graphical abstract

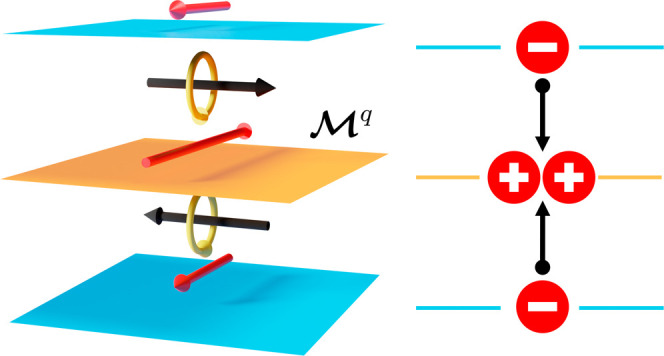

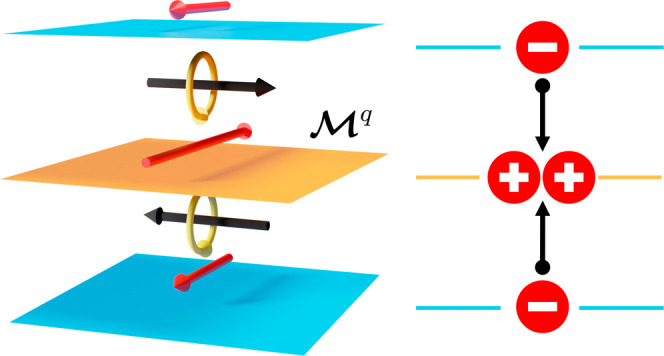

Moiré superlattices of 2D materials formed by lattice mismatch or misorientation have emerged as versatile platforms for exploring various exciting physics phenomena. Celebrated groundbreaking discoveries include superconductivity in magic-angle twisted bilayer graphene (TBG)^1^ and fractional quantum anomalous Hall effects in twisted bilayer MoTe_2_,^2–5^ which are made possible by the presence of flat energy bands boosting electron interactions. The chiral structure of twisted materials also leads to interesting effects that do not require strong electron interaction, for example, chiral optical and transport responses.^6–25^ It has been shown that twisted homobilayers with strong chiral interlayer coupling (e.g., TBG and twisted MoTe_2_) can support large counterflowing linear Hall currents in the two layers in the presence of time-reversal symmetry (TRS),^8,9,12,16,17^ leading to the layer Hall effect (LHE) beyond magnetic systems.^26–34^ In twisted bilayers, the layer Hall counterflows yield an in-plane magnetic dipole moment, underlying the emergence of circular dichroism in the quasi-2D limit. The Hall counterflows in a twisted bilayer can also be regarded as an interlayer electric dipole Hall current.^16^ In multilayer structures, the layer-dependent Hall currents are expected to exhibit richer patterns among different layers,^21,35^ leading to, e.g., interlayer electric quadrupole Hall currents and in-plane magnetic quadrupole moments in trilayers (Figure 1a). Here we explore such a LHE in the presence of TRS, by employing twisted trilayer graphene (TTG) as a model system. In mirror-symmetric TTG where the two twisted interfaces have opposite rotations,^36–39^ the LHE is manifested as a pure interlayer electric quadrupole Hall effect, as the dipole contribution is prohibited by the chiral structure. Interestingly, the charge Hall flows in the two outer layers have the same direction and magnitude, and the compensation by the hidden flow in the middle layer ensures that the overall charge Hall current vanishes. When an interlayer translation breaks the out-of-plane mirror symmetry, a pure electric quadrupole Hall effect is preserved, whose magnitude can be tuned by the translation when the twist angle is small. In large-angle twisted TTG, as interlayer coupling becomes weak at low energies, we demonstrate that the layer-resolved Hall currents can be accurately obtained by adding the contributions from the two individual twisted interfaces, which are modestly dependent on interlayer translation. Additionally, the layer Hall current contributed by the large-angle twisted interface follows a universal scaling behavior with respect to the twist angle and Fermi energy. These properties enable tailor-made layer-resolved Hall responses, or, equivalently, electric multipole Hall responses, in general twisted multilayers with arbitrary and possibly incommensurate twist angles.

**Figure 1:**
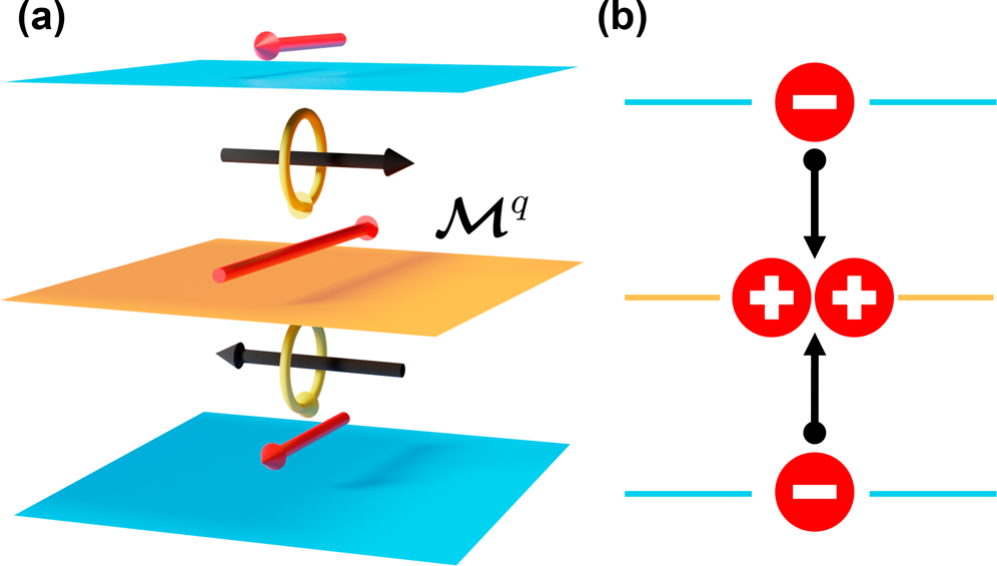
Schematics of layer-dependent Hall effects in a mirror-symmetric trilayer. (a) The Hall currents in the top and bottom layers are identical (shorter red arrows), while the current in the middle layer flows oppositely with a doubled magnitude (longer red arrow). (b) Such layer-dependent current flows correspond to the Hall current of interlayer electric quadrupole, which also generates an in-plane magnetic quadrupole moment composed of two opposite in-plane magnetic dipole moments contributed by current counterflows in adjacent layers. The yellow ring with a black arrow indicates the direction of the in-plane magnetic dipole at each twisted interface.

## Interlayer Electric Multipole Hall Effect and In-Plane Magnetic Multipole

For a multilayer system, the current in a specific layer *l* can be calculated by^16,31^

1 $$j^{l}=\frac{e}{4\pi^{2}}\underset{n}{\sum }\int f_{n}\left(k\right)v_{n}^{l}\left(k\right)\;dk$$

where *e* < 0 is the charge of an electron, *f*_*n*_ is the Fermi–Dirac distribution function on the *n*th band with energy *ε*_*n*_(**k**) and Bloch state |ψ_*n* **k**_⟩ = e^*i* **k**·**r**^|*u*_*n* **k**_⟩, and $v_{n}^{l}\left(k\right)=\left\langle u_{nk}\left|{\mathrm{v̂}}^{l}\right|u_{nk}\right\rangle =\left\langle u_{nk}\left|\frac{1}{2}\left\{\mathrm{v̂},P_{l}\right\}\right|u_{nk}\right\rangle$ is the velocity projected onto layer *l* with *P*_*l*_ = |*l*⟩⟨*l*|. Within the Boltzmann transport theory and the constant relaxation time τ approximation, the distribution function reads $f_{n}\approx f_{eq}-\frac{e}{\hbar }\tau E\cdot \partial_{k}f_{eq}$ to the linear order, where **E** is the applied electric field and *f*_eq_ is the equilibrium Fermi–Dirac distribution. We will focus on time-reversal symmetric systems and linear responses in the following. In such scenarios, only band velocity $v_{n}\left(k\right)=\left\langle u_{nk}\left|\mathrm{v̂}\right|u_{nk}\right\rangle =\hbar^{-1}\left\langle u_{nk}\left|\partial_{k}Ĥ\left(k\right)\right|u_{nk}\right\rangle$ can contribute to a non-zero current, and the transverse conductivity in layer *l* becomes

2 $$\sigma_{ij}^{l}=-\frac{e^{2}}{h}\frac{\hbar \tau }{2\pi }\underset{n}{\sum }\int \frac{\partial f_{eq}}{\partial \varepsilon_{n}}v_{n,i}^{l}\left(k\right)v_{n,j}\left(k\right)\;dk$$

where *i* ≠ *j* ∈ {*x*, *y*}. Then the Hall conductivity of layer *l*, defined by $\sigma_{H}^{l}=\frac{1}{2}\left(\sigma_{xy}^{l}-\sigma_{yx}^{l}\right)$ , reads

3 $$\sigma_{H}^{l}=\frac{e^{2}}{h}\frac{\hbar \tau }{4\pi }\underset{n}{\sum }\int \frac{\partial f_{eq}}{\partial \varepsilon_{n}}{\left[v_{n}\left(k\right)\times v_{n}^{l}\left(k\right)\right]}_{z}\;dk$$

and the corresponding Hall current is $j_{H}^{l}=\sigma_{H}^{l}ẑ\times E$ . In this work, we will focus on trilayer systems and comment on the generalization to more complicated multilayers at the end. In the case of TTG models considered in the following, the band velocity operators have simple diagonal structures in the layer space: $\mathrm{v̂}={\mathrm{v̂}}^{t}+{\mathrm{v̂}}^{m}+{\mathrm{v̂}}^{b}$ , where ${\mathrm{v̂}}^{t}=\mathrm{diag}\left({\mathrm{v̂}}_{1},0,0\right)$ , ${\mathrm{v̂}}^{m}=\mathrm{diag}\left(0,{\mathrm{v̂}}_{2},0\right)$ , and ${\mathrm{v̂}}^{b}=\mathrm{diag}\left(0,0,{\mathrm{v̂}}_{3}\right)$ . *t*, m, and b indicate the top, middle, and bottom layers, respectively, and ${\mathrm{v̂}}_{1∼3}$ can be easily identified once the Hamiltonian is explicitly specified.

The layer-dependent Hall currents in a multilayer can also be understood as the Hall currents of interlayer electric multipoles.^35^ For trilayer systems, one can define the interlayer electric dipole and quadrupole operators as ${Ê}_{⊥}^{d}=\mathrm{diag}\left(1,0,-1\right)$ and ${Ê}_{⊥}^{q}=\mathrm{diag}\left(1,-2,1\right)$ , the latter of which is schematically shown in Figure 1b. The corresponding electric dipole and quadrupole Hall currents read $j_{H}^{d,q}=\sigma_{H}^{d,q}ẑ\cdot E$ , where

4 $$\sigma_{H}^{d/q}=\frac{e^{2}}{h}\frac{\hbar \tau }{4\pi }\underset{n}{\sum }\int \frac{\partial f_{eq}}{\partial \varepsilon_{n}}{\left[v_{n}\left(k\right)\times v_{n}^{d/q}\left(k\right)\right]}_{z}\;dk$$

is the electric dipole/quadrupole Hall conductivity, with $v_{n}^{d/q}\left(k\right)=\left\langle u_{nk}\left|{\mathrm{v̂}}^{d/q}\right|u_{nk}\right\rangle$ being the dipole/quadrupole velocity:

5 $$\begin{array}{ll} {\mathrm{v̂}}^{d} & =\frac{1}{2}\left\{\mathrm{v̂},{Ê}_{⊥}^{d}\right\}=\left(\begin{array}{lll} {\mathrm{v̂}}_{1} & 0 & 0\\ 0 & 0 & 0\\ 0 & 0 & -{\mathrm{v̂}}_{3} \end{array}\right)\\ {\mathrm{v̂}}^{q} & =\frac{1}{2}\left\{\mathrm{v̂},{Ê}_{⊥}^{q}\right\}=\left(\begin{array}{lll} {\mathrm{v̂}}_{1} & 0 & 0\\ 0 & -2{\mathrm{v̂}}_{2} & 0\\ 0 & 0 & {\mathrm{v̂}}_{3} \end{array}\right) \end{array}$$

The electric dipole and quadrupole Hall conductivities are related to the layer Hall conductivities (eq 3) by

$$\begin{array}{llll} \sigma_{H}^{t} & +\sigma_{H}^{m}+\sigma_{H}^{b}=0 & & \sigma_{H}^{t}=\frac{1}{6}\sigma_{H}^{q}+\frac{1}{2}\sigma_{H}^{d}\\ \sigma_{H}^{d} & =\sigma_{H}^{t}-\sigma_{H}^{b}\Leftrightarrow & & \sigma_{H}^{m}=-\frac{1}{3}\sigma_{H}^{q}\\ \sigma_{H}^{q} & =\sigma_{H}^{t}+\sigma_{H}^{b}-2\sigma_{H}^{m} & & \sigma_{H}^{b}=\frac{1}{6}\sigma_{H}^{q}-\frac{1}{2}\sigma_{H}^{d} \end{array}$$

where $\sigma_{H}^{t}+\sigma_{H}^{m}+\sigma_{H}^{b}=0$ corresponds to a vanishing net linear charge Hall response in the presence of TRS as dictated by the Onsager reciprocal relation.^40^ One identifies that an interlayer electric dipole (quadrupole) Hall response implies $\sigma_{H}^{t}\neq \sigma_{H}^{b}$ $\left(\sigma_{H}^{m}\neq 0\right)$ or vice versa. A non-zero electric dipole Hall response requires the system to be chiral without inversion and mirror symmetries.^16^ Thus, twisted trilayers with out-of-plane mirror symmetry are ideal platforms for studying the pure electric quadrupole Hall effect.

Meanwhile, the presence of layer-dependent Hall currents gives rise to magnetic dipole and quadrupole moments, which might be probed by X-ray magnetic circular dichroism.^41,42^ Due to the infinite extension of the current distribution in the *x–y* plane, only the in-plane magnetic dipole and the magnetic quadrupole associated with the *z* coordinate are physically well-defined. This necessitates the definition of in-plane magnetic dipole and quadrupole vector densities, $M^{d}$ and $M^{q}$ , by expanding magnetic coupling energy C=−∫j·Adz between the in-plane current and an in-plane magnetic field with a weak *z* gradient and extracting the dipole and quadrupole terms $C_{d}={-M}^{d}\cdot B$ and $C_{q}=-{\frac{1}{2}M}^{q}\cdot \partial_{z}B$ . Specifically

6 $$M^{d}=\underset{l}{\sum }z_{l}ẑ\cdot j_{H}^{l}$$

7 $$M^{q}=\underset{l}{\sum }z_{l}^{2}\left(ẑ\cdot j_{H}^{l}\right)$$

where *z*_*l*_ is the out-of-plane coordinate of layer *l* and the coordinate origin is placed in the middle layer. In the linear response regime, we obtain

8 $$M^{d}=-d_{0}\left(\sigma_{H}^{t}-\sigma_{H}^{b}\right)E=-d_{0}\sigma_{H}^{d}E$$

9 $$M^{q}=-d_{0}^{2}\left(\sigma_{H}^{t}+\sigma_{H}^{b}\right)E=-\frac{1}{3}d_{0}^{2}\sigma_{H}^{q}E$$

where *d*_0_ is the spacing between adjacent layers. One observes that the induced in-plane longitudinal magnetic dipole and quadrupole are compactly expressed by the electric dipole and quadrupole Hall conductivities, respectively.

For a semiquantitative estimation, we take electric field strength *E* = 10^6^ V/m, interlayer spacing *d*_0_ = 3.35 Å, and Hall conductivity $\sigma_{H}^{d}∼\sigma_{H}^{q}∼10e^{2}/h$ , and then the in-plane magnetic dipole and quadrupole can be as large as $M^{d}∼10^{-2}{\;\mu }_{B}/{\mathrm{nm}}^{2}$ and $M^{q}∼10^{-3}{\;\mu }_{B}/\mathrm{nm}$ , where μ_B_ is the Bohr magneton.

## Electric Quadrupole Hall Effect in Symmetrically Twisted Trilayer Graphene

Symmetrically twisted trilayers are ideal platforms for studying the pure electric quadrupole Hall effect as the dipole Hall effect is suppressed by symmetry. The interlayer translation and twist angles between adjacent layers also allow tuning of the magnitude of the currents.

### Mirror-Symmetric Twisted Trilayer Graphene

We first consider mirror-symmetric TTG, where the outer layers are aligned and rotated with respect to the middle layer by angle θ [Figure 3(d)]. The mirror-symmetric TTG Hamiltonian in the + *K* valley reads^36,37^

10 $$\begin{array}{ll} & H_{mTTG}=\\ & \left(\begin{array}{lll} h_{0}\left(K_{t},\frac{\theta }{2}\right) & U_{12} & 0\\ U_{12}^{†} & h_{0}\left(K_{m},-\frac{\theta }{2}\right) & U_{23}^{†}\\ 0 & U_{23} & h_{0}\left(K_{b},\frac{\theta }{2}\right) \end{array}\right) \end{array}$$

where $h_{0}\left(K,\theta \right)=-\hbar v_{F}\left(-i\nabla -K\right)\cdot R_{\theta }\left(-\sigma_{x},\sigma_{y}\right)$ describes the twisted Dirac cone, *R*_θ_ denotes the rotation matrix by angle θ, and *v*_F_ = 0.8 × 10^6^ m/s is the Fermi velocity of graphene. The Dirac points are located at $K_{t}=K_{b}=R_{\theta /2}K_{0}$ and $K_{m}=R_{-\theta /2}K_{0}$ in the three layers, where $K_{0}=\left(\frac{4\pi }{3a},0\right)$ and *a* = 2.46 Å. The interlayer coupling terms read $U_{12}=U_{23}=U_{0}+U_{1}e^{-iG_{1}\cdot r}+U_{2}e^{-i\left(G_{1}+G_{2}\right)\cdot r}$ , where $G_{1}=\frac{2\pi }{b}\left(\frac{1}{\sqrt{3}},1\right)$ , $G_{2}=\frac{2\pi }{b}\left(\frac{2}{\sqrt{3}},0\right)$ , $b=\frac{a}{2\sin\left(\theta /2\right)}$ , and $U_{n}=\left(\begin{array}{ll} u_{AA} & u_{AB}e^{i2\pi /3n}\\ u_{AB}e^{-i2\pi /3n} & u_{AA} \end{array}\right)$ , with *u*_AA_ = 79.7 meV and *u*_AB_ = 97.5 meV.^43^

Figure 2 shows the energy bands of *H*_mTTG_ and the corresponding layer-resolved Hall conductivities under two different twist angles. In the band structure, there is a Dirac cone at the κ′ point uncoupled from other bands due to out-of-plane mirror symmetry *M*_*h*_,^36,37^ which can be understood as follows. The basis of the trilayer’s Bloch states can be categorized according to the eigenvalue of *M*_*h*_: two even states |m⟩, |t⟩ + |b⟩ and one odd state |t⟩ – |b⟩. The *M*_*h*_-odd state is decoupled from the even ones, retaining the linear dispersion of the monolayers and has no contribution to the layer Hall currents. This can be explicitly shown by performing a transformation

11 $$Q=\frac{1}{\sqrt{2}}\left(\begin{array}{lll} 1 & 0 & 1\\ 0 & \sqrt{2} & 0\\ -1 & 0 & 1 \end{array}\right)$$

which changes the basis to states |t⟩ + |b⟩, |m⟩, and |t⟩ – |b⟩ via mixing |t⟩ and |b⟩ in the first and last line. For mirror-symmetric TTG, we have $U_{12}=U_{23}$ and **K**_t_ = **K**_b_, and then the transformed Hamiltonian is block diagonal:

12 $$\begin{array}{ll} & QH_{mTTG}Q^{†}=\\ & \left(\begin{array}{lll} h_{0}\left(K_{t},\frac{\theta }{2}\right) & \sqrt{2}U_{12} & 0\\ \sqrt{2}U_{12}^{†} & h_{0}\left(K_{m},-\frac{\theta }{2}\right) & 0\\ 0 & 0 & h_{0}\left(K_{t},\frac{\theta }{2}\right) \end{array}\right) \end{array}$$

**Figure 2:**
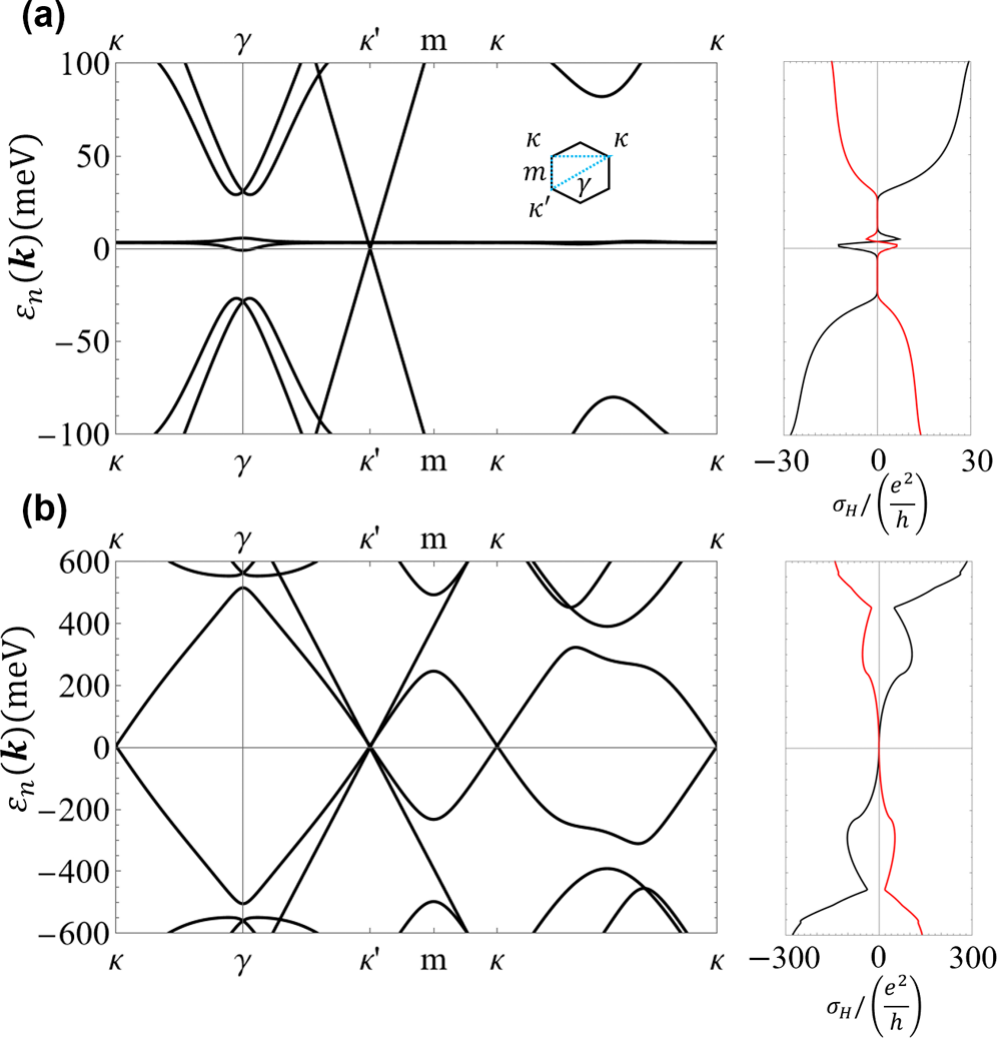
Moiré bands (left) and layer Hall conductivities (right) of mirror-symmetric TTG with twist angles of (a) 1.5° and (b) 5°. In the conductivity plots, the red curves are for the outer layers and the black curves are for the middle layer. The inset in panel a illustrates the Brillouin zone and the high-symmetry points. τ = 1 ps throughout this work.

The top-left 2 × 2 block is equivalent to the model of TBG^43,44^ with enlarged interlayer tunneling, which is responsible for the layer Hall currents. Note the Dirac point energy from this effective TBG model (e.g., at κ in Figure 2) is slightly higher than zero due to interlayer coupling.^37^ The Hall currents in the top and bottom layers are equal, ensured by the *M*_*h*_ symmetry; the Hall current in the middle layer flows oppositely with doubled magnitude, ensuring a null net Hall current from the trilayer by the Onsager relation.^21^ The Hall conductivity of the layer can be very large (e.g., 10 ∼ 100*e* ^2^/*h* with a relaxation time of τ = 1 ps), and its profile is similar to that of TBG^8,9,12,16,17^ as expected, which is nearly antisymmetric along the energy axis and peaks at energies with strong interlayer coupling. Overall, the Hall currents in the three layers constitute an interlayer electric quadrupole Hall current, and the resultant opposite in-plane magnetic dipole moments contributed by the two twisted interfaces lead to an in-plane magnetic quadrupole moment (Figure 1).

### Effects of Interlayer Translation

Keeping the twisted angle unchanged, the electronic properties of TTG can be tuned by the lateral shift between the two outer layers. Starting from a mirror-symmetric TTG, we assume that the top layer is translated by **δ** with respect to the bottom layer (Figure 3d). The translation enters the Hamiltonian through the interlayer coupling between the top and middle layers. To see this, it is convenient to rewrite the interlayer tunneling term as $U_{12}=U_{0}+U_{1}e^{-ib_{1}\cdot d}+U_{2}e^{-i\left(b_{1}+b_{2}\right)\cdot d}$ , where $d=\left(R_{\theta /2}-R_{-\theta /2}\right)r$ is the local interlayer displacement vector due to rotation. When the top layer is translated by **δ**, the local interlayer displacement vector becomes **d** + **δ**; thus, the interlayer coupling between the top and middle layers becomes ${Ũ}_{12}=U_{0}+U_{1}e^{-ib_{1}\cdot \delta }e^{-iG_{1}\cdot r}+U_{2}e^{-i\left(b_{1}+b_{2}\right)\cdot \delta }e^{-i\left(G_{1}+G_{2}\right)\cdot r}$ . The effects of top-layer translation are thus encoded in the two extra phases. In the following, we parametrize **δ** = (η_1_, η_2_) = η_1_ **a**_1_ + η_2_ **a**_2_, where η_1,2_ ∈ [0, 1] are some constants (Figure 3d). Note that although the *M*_*h*_ symmetry is broken by the translation, the *C*_2_ *M*_*h*_ symmetry is respected;^45^ thus, the Hall currents in the outer layers remain identical, whose sum is canceled by the Hall current in the middle layer. Therefore, in the translated TTG, the electric dipole Hall effect is still forbidden, and the layer Hall currents manifest as a pure quadrupole Hall effect.

**Figure 3:**
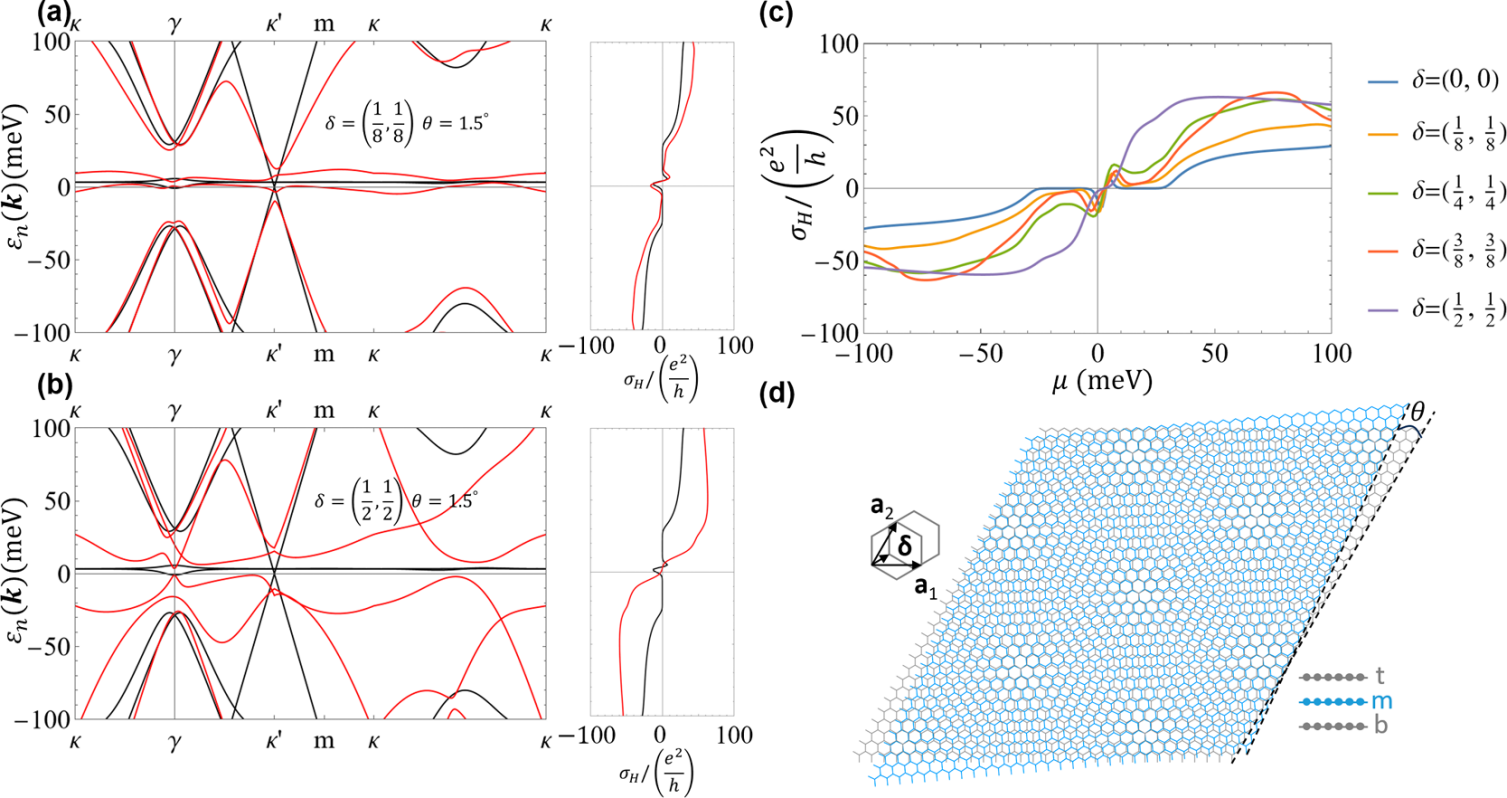
Moiré bands (left) and middle-layer Hall conductivity (right) of top-layer-translated TTG with translation η_1_ = η_2_ = (a) 1/8 and (b) 1/2. The black curves are for zero translation, and the red curves are for non-zero translations. (c) Middle-layer Hall conductivity of different translations and chemical potentials. The twist angle is fixed at 1.5° in panels a–c. (d) Schematics of a mirror-symmetric twisted trilayer, where the top and bottom layers (gray) are aligned. The left inset illustrates the top-layer translation with **δ** = η_1_ **a**_1_ + η_2_ **a**_2_.

The red curves in panels a and b of Figure 3 present the energy bands and the middle-layer Hall conductivity of TTG with a small twist angle (1.5°) and two different top-layer translations. As the translation breaks the *M*_*h*_ symmetry, the *M*_*h*_-odd state |t⟩ – |b⟩ is coupled to the even states, opening a gap in the Dirac cone at the κ′ point. For small twist angles, the energy bands change dramatically due to this additional state entering the interlayer coupling. Correspondingly, the profile and magnitude of layer Hall conductivity are extremely sensitive to the value of translation (Figure 3c). One notices that the layer Hall conductivity generally increases with the translation. In contrast, for large twist angles, the effects of interlayer coupling are pronounced at much higher energies (Figure 4a), while the low-energy bands are barely affected by the translation (Figure 4b). Nevertheless, the layer Hall conductivity is sizable at low energies, and it is weakly affected by the interlayer translation, where a modest fluctuation can be observed only near the charge neutrality point (Figure 4c) due to the small gap openings at the κ′ point (Figure 4d). The weak interlayer coupling and translation independence at low energies in large-angle TTG make it possible to extend the above discussions to general multilayers consisting of varying twist angles that do not even have a well-defined periodicity.

**Figure 4:**
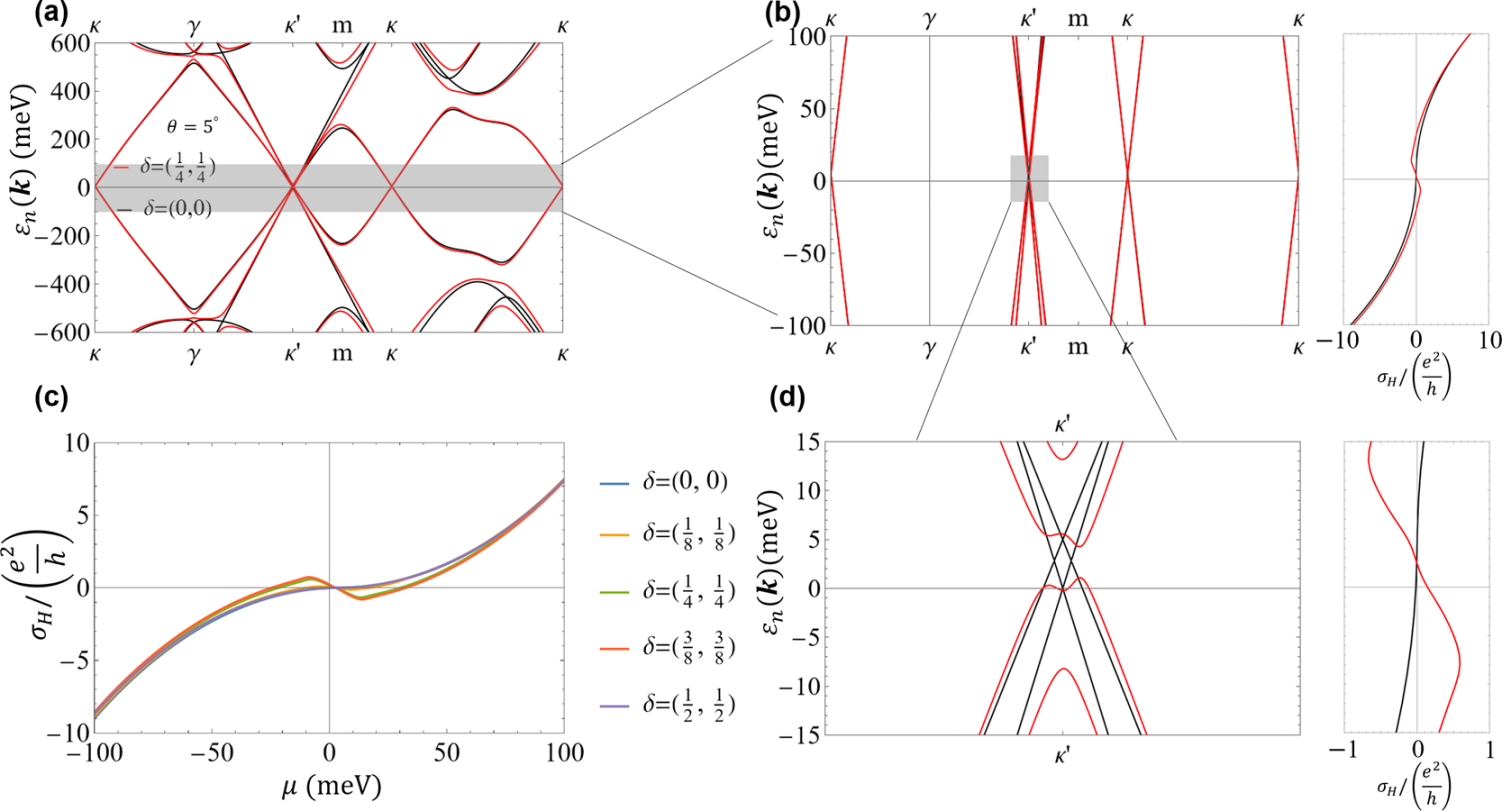
(a) Moiré bands of top-layer-translated TTG with translation δ = (1/4, 1/4) and a twist angle of 5°. The black curves are for zero translation, and the red curves are for non-zero translation. (b and d) Magnified moiré bands (left) and middle-layer Hall conductivity (right) in energy windows of [−100, 100] and [−15, 15] meV, respectively. (c) Middle-layer Hall conductivity of top-layer-translated TTG with different translations for a 5° twist in the energy window of [−100, 100] meV.

## Twisted Multilayer Graphene with Different Twist Angles: A Perturbative View

In large-angle TTG, the momentum mismatch of the low-energy valleys between adjacent layers is substantially larger than the interlayer coupling energy scale. Consequently, within the low-energy window near the charge neutrality point, the interlayer coupling acts as a weak perturbation.^16^ For the middle layer of a TTG system, the electronic states are subjected to two distinct moiré potentials originating from the top and bottom twisted interfaces. In this large-angle perturbative regime, higher-order scattering processes that cross-couple both interfaces can be safely ignored. Therefore, one can decouple the trilayer system and treat the physical quantities of the middle layer as a simple linear superposition of the independent contributions from the two adjacent TBG interfaces.

To examine this, we evaluate the middle-layer Hall conductivity of mirror-symmetric TTG in two ways: (i) by performing the calculation exactly using a full trilayer Hamiltonian and (ii) by adding the separated contributions from two individual TBG systems of opposite twist angles (Figure 5a, orange arrows). As shown in Figure 5c, the Hall conductivity of the middle layer for mirror-symmetric TTG with large twist angles θ ≳ 5° can be well approximated by the independent contributions from two TBG interfaces, i.e., $\sigma_{H}^{m}\left(\theta ,-\theta \right)\approx {\mathrm{\sigma ̃}}_{H}^{b}\left(\theta \right)+{\mathrm{\sigma ̃}}_{H}^{t}\left(-\theta \right)$ , where θ and −θ denote the twist angles at the bottom and top interfaces, respectively. Here, ${\mathrm{\sigma ̃}}_{H}^{l}\left(\theta \right)$ denotes the Hall conductivity in layer *l* of the TBG with twist angle θ, satisfying ${\mathrm{\sigma ̃}}_{H}^{t}\left(\theta \right)=-{\mathrm{\sigma ̃}}_{H}^{b}\left(\theta \right)=-{\mathrm{\sigma ̃}}_{H}^{t}\left(-\theta \right)$ .^16^ Furthermore, we note that the layer Hall conductivity at large twist angles exhibits a robust scaling behavior: $\sigma_{H}^{m}\left(\theta ,-\theta \right)\approx 2{\mathrm{\sigma ̃}}_{H}^{b}\left(\theta \right)\approx \theta^{-3}f\left(\mu \right)e^{2}/h$ , where *f*(μ) = *C*(μ – μ_0_)|μ – μ_0_| with fitting parameters *C* = 3.426 × 10^–6^ meV^–2^ and μ_0_ = 2.747 meV. Small offset μ_0_ originates from the slight shift of the charge neutrality point due to the weak interlayer coupling.

**Figure 5:**
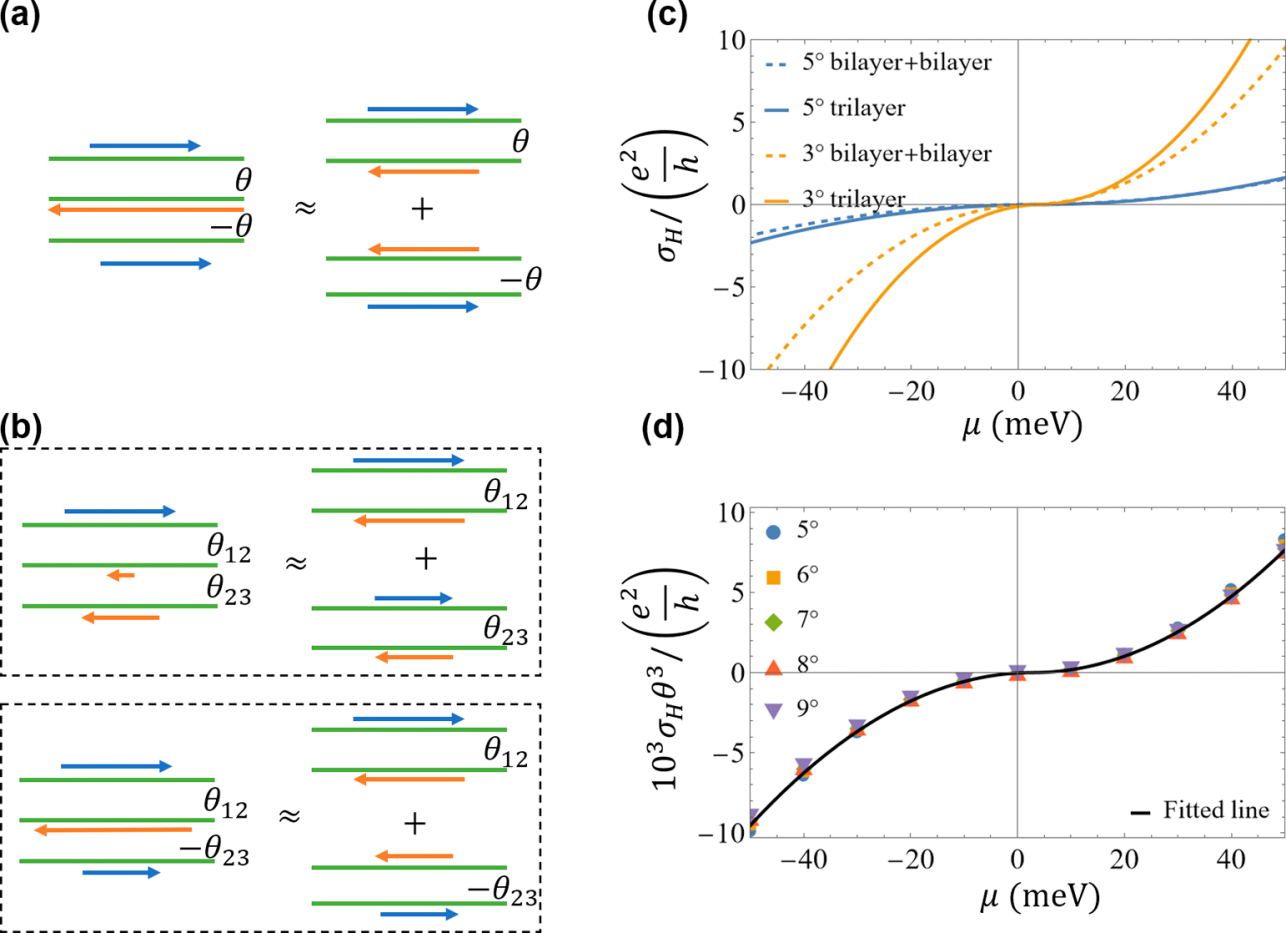
(a) Schematics for perturbative calculation of layer-resolved Hall currents in mirror-symmetric TTG. (b) Schematics for perturbative approximation of layer-resolved Hall currents in TTG with general twist angles: (top) helical twisted trilayer and (bottom) alternating twisted trilayer. Here θ_12_, θ_23_ > 0. (c) Middle-layer Hall conductivities of TTG with twist angles of 3° (orange) and 5° (blue). The solid curves are from direct calculation of a trilayer, and the dashed curves are calculated by adding up the contributions of two interfaces of TBG. (d) Middle-layer Hall conductivities of mirror-symmetric TTG with larger twist angles (scattered symbols), which exhibits a θ^3^ scaling. The black curve is the fitted curve *f*(μ) (see the text). The unit of θ is radians.

The validity of this linear additive rule in TTG provides a powerful framework for generalization to more complex multilayer geometries. As long as all adjacent twist angles remain in the large-angle perturbative regime, the absence of cross-interface coupling allows us to completely decompose the multilayer system into a series of decoupled TBG interfaces. This feature naturally extends our analysis to multilayer systems containing diverse twist-angle combinations, explicitly bypassing the computational difficulty of the multilayer with arbitrary twist-angle combination from the absence of long-range periodicity. As an example of the simplest multilayer, Figure 5b schematically illustrates helical TTG (top)^46–48^ and alternating TTG (bottom), demonstrating how the total layer-resolved response can be straightforwardly assembled from the relevant isolated twisted bilayer contributions.

In this work, we focus on exploring the effects of the twist angle and interlayer translation on the layer-dependent Hall currents in TTG. In alternating TTG with opposite twist angles θ and −θ at the two twisted interfaces, we show that the Hall currents in the top and bottom layers are identical, while the middle layer holds an opposite Hall current of a doubled amplitude (Figure 1). This specific layer-dependent Hall current configuration also corresponds to a pure interlayer electric quadrupole Hall current and generates an in-plane magnetic quadrupole moment. When θ is small, interlayer translation can affect the layer Hall currents dramatically, serving as a tuning knob for controlling the magnitude of the currents. When θ is large, interlayer translation has minor effects on the magnitude of the layer Hall currents at low energies. Importantly, in this large-angle regime the layer Hall currents in the TTG can be accurately reproduced by adding the contributions from the two individual twisted interfaces (Figure 5a). We also identify a universal scaling behavior of the layer Hall current contributed by large-angle twisted interfaces with respect to twist angle θ and Fermi energy. This makes it possible to obtain the layer Hall currents in generic multilayers with twisted interfaces of varying angles by decomposing the multilayer stack into a series of individual bilayer interfaces and summing over their layer Hall currents (Figure 5b). The layer Hall currents can be further tuned by applying an interlayer displacement field. Due to the breaking of *M*_*h*_ and *C*_2_ *M*_*h*_ symmetries, the Hall currents in the top and bottom layers will be different, pointing to the presence of an interlayer electric dipole Hall effect and in-plane magnetic dipole moment in addition to their quadrupole counterparts (see section S1 of the Supporting Information for details). Experimentally, the layer-dependent Hall currents can be probed with layer-resolved measurements,^16,49^ the details of which are discussed in section S2. Our work shows that twisted van der Waals materials are promising platforms for exploring layertronics responses with rich internal structures. Our findings could stimulate future studies on understanding fundamental magnetic properties of quasi-2D systems^31,50,51^ and seeking potential applications of twisted materials for magnetoelecctric effects.^52,53^

## Supplementary Material



## Notes

Details about the dipole Hall effect in TTG with displacement field and discussion about experimental detection schemes (PDF)

## References

[1] Cao Y., Fatemi V., Fang S., Watanabe K., Taniguchi T., Kaxiras E., Jarillo-Herrero P. (2018). Unconventional superconductivity
in magic-angle graphene superlattices. Nature.

[2] Cai J. (2023). Signatures of fractional
quantum anomalous Hall states in twisted
MoTe2. Nature.

[3] Zeng Y., Xia Z., Kang K., Zhu J., Knüppel P., Vaswani C., Watanabe K., Taniguchi T., Mak K. F., Shan J. (2023). Thermodynamic evidence
of fractional
Chern insulator in moiré MoTe2. Nature.

[4] Park H. (2023). Observation of fractionally
quantized anomalous Hall effect. Nature.

[5] Xu F. (2023). Observation of Integer
and Fractional Quantum Anomalous Hall Effects
in Twisted Bilayer MoTe_2_. Phys. Rev.
X.

[6] Kim C.-J., Sánchez-Castillo A., Ziegler Z., Ogawa Y., Noguez C., Park J. (2016). Chiral atomically
thin films. Nat. Nanotechnol..

[7] Suárez
Morell E., Chico L., Brey L. (2017). Twisting dirac fermions:
circular dichroism in bilayer graphene. 2D Mater..

[8] Stauber T., Low T., Gómez-Santos G. (2018). Chiral Response
of Twisted Bilayer
Graphene. Phys. Rev. Lett..

[9] Stauber T., Low T., Gómez-Santos G. (2018). Linear response
of twisted bilayer
graphene: Continuum versus tight-binding models. Phys. Rev. B.

[10] Addison Z., Park J., Mele E. J. (2019). Twist,
slip, and circular dichroism
in bilayer graphene. Phys. Rev. B.

[11] Ochoa H., Asenjo-Garcia A. (2020). Flat Bands
and Chiral Optical Response of Moiré
Insulators. Phys. Rev. Lett..

[12] Stauber T., González J., Gómez-Santos G. (2020). Change of chirality at magic angles
of twisted bilayer graphene. Phys. Rev. B.

[13] Bahamon D. A., Gómez-Santos G., Stauber T. (2020). Emergent magnetic texture in driven
twisted bilayer graphene. Nanoscale.

[14] Stauber T., Low T., Gómez-Santos G. (2020). Plasmon-Enhanced
Near-Field Chirality
in Twisted van der Waals Heterostructures. Nano
Lett..

[15] Liu Y., Holder T., Yan B. (2021). Chirality-Induced Giant Unidirectional
Magnetoresistance in Twisted Bilayer Graphene. Innovation.

[16] Zhai D., Chen C., Xiao C., Yao W. (2023). Time-reversal even
charge hall effect from twisted interface coupling. Nat. Commun..

[17] Zhu J., Zhai D., Xiao C., Yao W. (2024). Layer Hall counterflow
as a model probe of magic-angle twisted bilayer graphene. Phys. Rev. B.

[18] Chen C., Zhai D., Xiao C., Yao W. (2024). Crossed nonlinear dynamical
Hall effect in twisted bilayers. Phys. Rev.
Res..

[19] Li J., Zhai D., Xiao C., Yao W. (2024). Dynamical chiral Nernst
effect in twisted Van der Waals few layers. Quantum Front..

[20] Hu J.-X., Zeng C., Yao Y. (2024). Colossal layer
Nernst effect in twisted
moiré layers. Phys. Rev. B.

[21] Margetis D., Gómez-Santos G., Stauber T. (2024). Optical response of alternating twisted
trilayer graphene. Phys. Rev. B.

[22] Li C., Yao W. (2024). Chiral excitonic systems
in twisted bilayers from Förster
coupling and unconventional excitonic Hall effects. Phys. Rev. B.

[23] Okyay M. S., Choi M., Xu Q., Diéguez A. P., Del Ben M., Ibrahim K. Z., Wong B. M. (2025). Unconventional nonlinear
Hall effects in twisted multilayer 2D materials. npj 2D Mater. Appl..

[24] Okyay M. S., Sato S. A., Kim K. W., Yan B., Jin H., Park N. (2022). Second harmonic Hall responses of
insulators as a probe of Berry
curvature dipole. Commun. Phys..

[25] Kuhn M. H. G., Silva L. A., Bahamon D. A. (2025). Layer-resolved
quantum transport
in twisted bilayer graphene: Counterflow and machine learning predictions. Phys. Rev. B.

[26] Gao A. (2021). Layer Hall effect in
a 2D topological axion antiferromagnet. Nature.

[27] Xu H., Zhou J., Li J. (2021). Light-Induced
Quantum Anomalous Hall
Effect on the 2D Surfaces of 3D Topological Insulators. Adv. Sci..

[28] Chen R., Sun H.-P., Gu M., Hua C.-B., Liu Q., Lu H.-Z., Xie X. C. (2024). Layer Hall effect induced by hidden
Berry curvature in antiferromagnetic insulators. Natl. Sci. Rev..

[29] Dai W.-B., Li H., Xu D.-H., Chen C.-Z., Xie X. C. (2022). Quantum anomalous
layer Hall effect in the topological magnet MnBi_2_Te_4_. Phys. Rev. B.

[30] Li S., Gong M., Cheng S., Jiang H., Xie X. C. (2024). Dissipationless
layertronics in axion insulator MnBi2Te4. Natl.
Sci. Rev..

[31] Fan F.-R., Xiao C., Yao W. (2024). Intrinsic
dipole Hall effect in twisted
MoTe2: magnetoelectricity and contact-free signatures of topological
transitions. Nat. Commun..

[32] Wang X., Liu S., Bai L., Zhang R.-W., Yao Y., Feng W. (2025). Layer Hall
and layer spin Hall effects in two-dimensional altermagnets induced
by spin-layer coupling. Phys. Rev. B.

[33] Han Y., Guo Y., Li Z., Qiao Z. (2025). Layer Hall Effect without External
Electric Field. Phys. Rev. Lett..

[34] Du W., Dou K., Li X., Dai Y., Wang Z., Huang B., Ma Y. (2025). Topological layer Hall
effect in two-dimensional type-I multiferroic
heterostructure. Nat. Commun..

[35] Zheng H., Zhai D., Xiao C., Yao W. (2024). Interlayer Electric
Multipoles Induced by In-Plane Field from Quantum Geometric Origins. Nano Lett..

[36] Khalaf E., Kruchkov A. J., Tarnopolsky G., Vishwanath A. (2019). Magic angle
hierarchy in twisted graphene multilayers. Phys.
Rev. B.

[37] Carr S., Li C., Zhu Z., Kaxiras E., Sachdev S., Kruchkov A. (2020). Ultraheavy
and Ultrarelativistic Dirac Quasiparticles in Sandwiched Graphenes. Nano Lett..

[38] Park J. M., Cao Y., Watanabe K., Taniguchi T., Jarillo-Herrero P. (2021). Tunable strongly
coupled superconductivity in magic-angle twisted trilayer graphene. Nature.

[39] Hao Z., Zimmerman A. M., Ledwith P., Khalaf E., Najafabadi D. H., Watanabe K., Taniguchi T., Vishwanath A., Kim P. (2021). Electric field–tunable superconductivity in alternating-twist
magic-angle trilayer graphene. Science.

[40] Onsager L. (1931). Reciprocal
Relations in Irreversible Processes. I. Phys.
Rev..

[41] Sasabe N., Ishii Y., Yamasaki Y. (2025). X-ray magnetic circular dichroism
originating from the anisotropic magnetic dipole operator in collinear
altermagnets under trigonal crystal field. Phys.
Rev. B.

[42] Bedoya-Pinto A., Ji J.-R., Pandeya A. K., Gargiani P., Valvidares M., Sessi P., Taylor J. M., Radu F., Chang K., Parkin S. S. P. (2021). Intrinsic 2D-XY
ferromagnetism in a van der Waals monolayer. Science.

[43] Koshino M., Yuan N. F. Q., Koretsune T., Ochi M., Kuroki K., Fu L. (2018). Maximally Localized Wannier Orbitals and the Extended Hubbard Model
for Twisted Bilayer Graphene. Phys. Rev. X.

[44] Bistritzer R., MacDonald A. H. (2011). Moiré
bands in twisted double-layer graphene. Proc.
Natl. Acad. Sci. U.S.A..

[45] Qin W., MacDonald A. H. (2021). In-Plane Critical Magnetic Fields in Magic-Angle Twisted
Trilayer Graphene. Phys. Rev. Lett..

[46] Zhao P., Xiao C., Yao W. (2021). Universal
superlattice potential
for 2D materials from twisted interface inside h-BN substrate. npj 2D Mater. Appl..

[47] Devakul T., Ledwith P. J., Xia L.-Q., Uri A., de la Barrera S. C., Jarillo-Herrero P., Fu L. (2023). Magic-angle helical trilayer graphene. Sci. Adv..

[48] Xia L.-Q., de la Barrera S. C., Uri A., Sharpe A., Kwan Y. H., Zhu Z., Watanabe K., Taniguchi T., Goldhaber-Gordon D., Fu L., Devakul T., Jarillo-Herrero P. (2025). Topological bands and correlated
states in helical trilayer graphene. Nat. Phys..

[49] Cao L. W., Wu C., Lyu L., Cohen L., Samuelson N., Yan Z., Pancholi S., Watanabe K., Taniguchi T., Parker D. E., Young A. F., Allen M. T. (2026). Layer-Resolved Microwave
Imaging of a van der Waals Heterostructure. Nano Lett..

[50] Zheng H., Zhai D., Xiao C., Yao W. (2025). Layer Coherence Origin
of Planar Hall Effect: From Charge to Multipole and Valley. Nano Lett..

[51] Hu J.-X., Sun Z.-T., Yao Y. (2026). Theory of In-Plane
Orbital Magnetization
with Layer Hybridization. arXiv.

[52] Spaldin N. A., Ramesh R. (2019). Advances in magnetoelectric
multiferroics. Nat. Mater..

[53] Fiebig M. (2005). Revival of
the magnetoelectric effect. J. Phys. D: Appl.
Phys..

