# Supplementary material for: Interlayer Electric Multipole Hall Effect in Twisted Multilayers
Source: Nano Lett. 2026 Jul 25;26(30):9783–90. doi: 10.1021/acs.nanolett.6c01563 (PMC13450481; doi:10.1021/acs.nanolett.6c01563)
Supplement: Supplementary file 1 [file nl6c01563_si_001.pdf]

Supporting Information for "*Interlayer electric multipole Hall effect in twisted multilayers*"

Chengxin Xiao,[†,‡,¶] Cong Xiao,[§,‖] Dawei Zhai,[*,†,¶,⊥] and Wang Yao[*,†,¶,⊥]

†*New Cornerstone Science Laboratory, Department of Physics, The University of Hong Kong, Hong Kong, China*

‡*School of Electrical and Information Engineering, Zhengzhou University, Zhengzhou, Henan 450001, China*

¶*HK Institute of Quantum Science & Technology, The University of Hong Kong, Hong Kong, China*

§*Interdisciplinary Center for Theoretical Physics and Information Sciences (ICTPIS), Fudan University, Shanghai 200433, China*

‖*State Key Laboratory of Surface Physics, Fudan University, Shanghai 200433, China*

⊥*State Key Laboratory of Optical Quantum Materials, The University of Hong Kong, Hong Kong, China*

E-mail: dzhai@hku.hk; wangyao@hku.hk

S1. Emergence of interlayer electric dipole Hall effect induced by displacement field

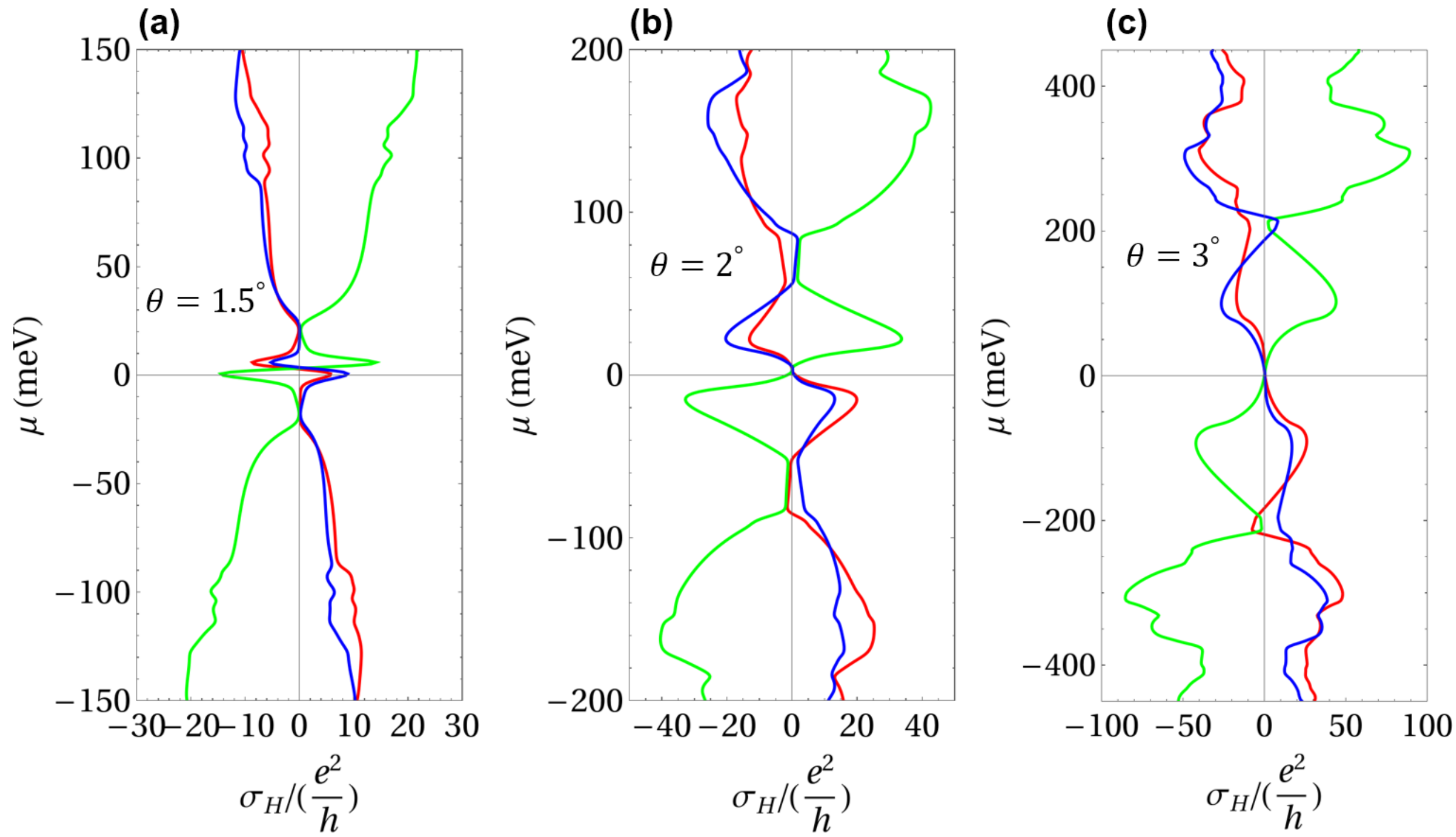

Figure S1: Layer Hall conductivity of mirror-symmetric TTG with twist angle (a) 1.5° (b) 2° (c) 3° and displacement field $eDd_0 = 50$meV. The red, blue and green curves represent top, middle and bottom layers, respectively.

Here we show the layer Hall effect when M_h and C_2M_h are both broken by an out-of-plane displacement field D. The displacement field enters the Hamiltonian as a layer-dependent onsite energy term:

$$H_D = \begin{pmatrix} eDd_0 & 0 & 0 \\ 0 & 0 & 0 \\ 0 & 0 & -eDd_0 \end{pmatrix}, \tag{1}$$

where $d_0 = 3.35\,\text{Å}$ is the interlayer spacing and the electrostatic potential is referenced to the middle layer. This term is odd under M_h and C_2M_h, thus breaking both M_h and C_2M_h simultaneously. The total Hamiltonian then reads $H_{\text{total}} = H_{\text{mTTG}}(\boldsymbol{\delta}) + H_D$, where

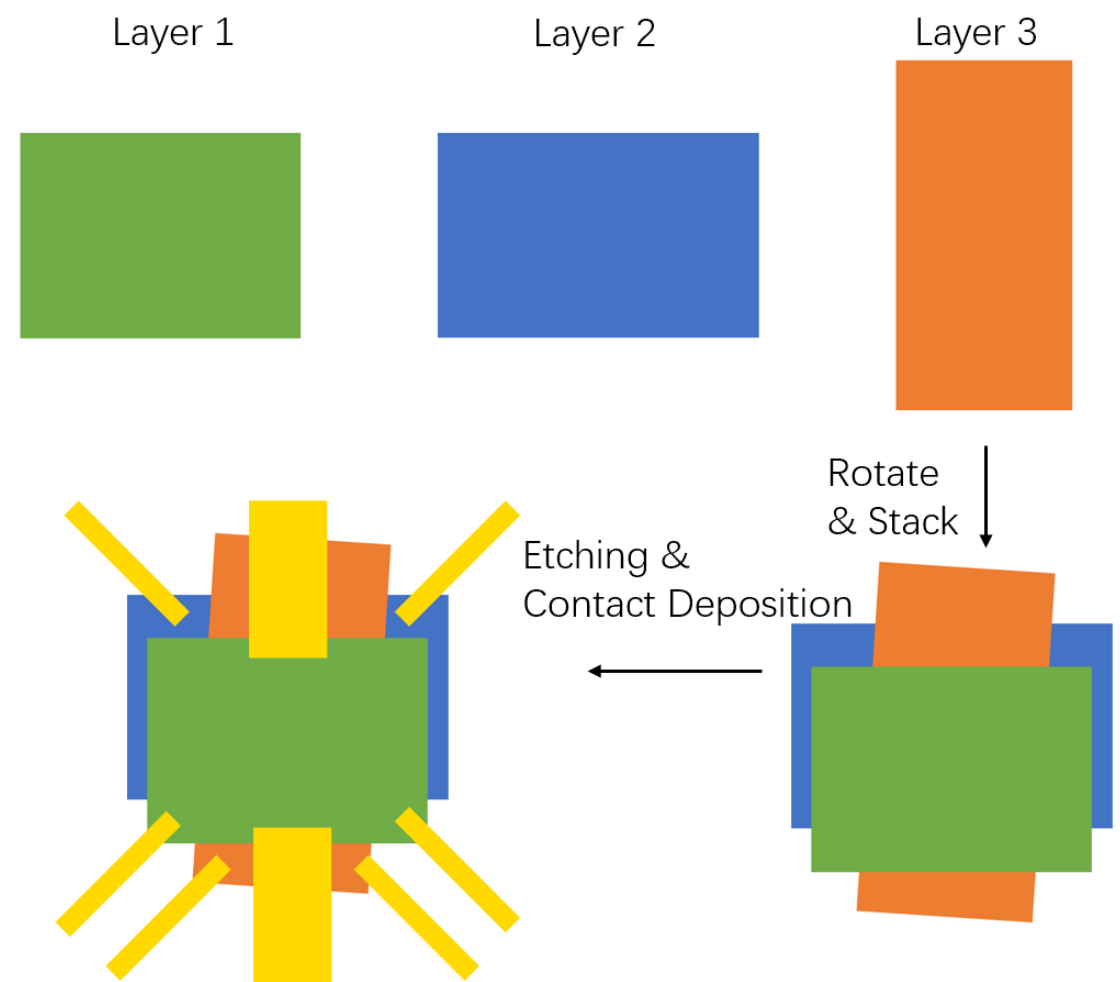

Figure S2: Schematics of stacking three monolayers with different sizes/shapes. Electrical contacts can be deposited onto the monolayer regions for layer-resolved Hall measurements.

$H_{\mathrm{mTTG}}(\boldsymbol{\delta})$ is the mirror-symmetric twisted trilayer graphene (TTG) Hamiltonian with top layer translation $\boldsymbol{\delta}$ introduced in the main text.

Fig. S1 shows the layer-resolved Hall conductivities σ_H^t, σ_H^m, and σ_H^b as function of the chemical potential for a few different twist angles in the mirror-symmetric case ($\boldsymbol{\delta} = 0$). As expected from the symmetry breaking, $\sigma_H^t \neq \sigma_H^b$ gives rise to a finite electric dipole Hall conductivity $\sigma_H^d = \sigma_H^t - \sigma_H^b$. These results demonstrate that an interlayer displacement field serves as an effective control knob to switch on the electric dipole Hall effect on top of the quadrupole background, enriching the magnetoelectric functionalities accessible in twisted multilayer systems.

S2. Experimental detection schemes

Layer-resolved Hall transport measurement—The interlayer electric quadrupole Hall effect is characterized by a specific $1 : -2 : 1$ ratio of Hall currents in the top, middle, and bottom layers. Its detection therefore can be done via layer-resolved electrical transport measurements that distinguish currents flowing in different layers.[1] Here we propose an experimental scheme based on a Hall bar geometry with individual layer contacts, as illustrated in Fig. S2.

The fabrication of electrical contacts to individual layers for layer-resolved measurement is experimentally feasible in coupled multilayer systems. Fig. S2 illustrates this by employing monolayer flakes with different sizes and/or shapes that are stacked and etched into a Hall bar geometry, where the three layers are contacted independently for Hall voltage measurements. For the pure electric quadrupole Hall effect in mirror-symmetric TTG, the expected Hall voltage ratio is $V_H^t : V_H^m : V_H^b = 1 : -2 : 1$, providing a unique experimental signature.

X-ray magnetic circular dichroism—The in-plane magnetic quadrupole moment could be detected via magneto-optical techniques. X-ray magnetic circular dichroism (XMCD) is a promising probe, as it combines element-specificity with high sensitivity to weak magnetic signals at surfaces and interfaces.[2] It has been employed to detect magnetic ordering in atomically thin van der Waals materials[3] and was proposed to probe magnetic quadrupole in altermagnets.[4] Although XMCD measurement of magnetic quadrupole in 2D materials has not been reported so far, our findings might motivate future experimental efforts.

References

(1) Zhai, D.; Chen, C.; Xiao, C.; Yao, W. Time-reversal even charge hall effect from twisted interface coupling. *Nat. Commun.* **2023**, *14*, 1961.

(2) Zhang, W.; Fu, Q.; Li, J.; Lian, B.; Xia, Y.; Zhou, L.; Wee, A. T. S.; Wong, P. K. J. Probing van der Waals magnetic surface and interface via circularly polarized X-rays. *Appl. Phys. Rev.* **2023**, *10*, 041308.

(3) Bedoya-Pinto, A.; Ji, J.-R.; Pandeya, A. K.; Gargiani, P.; Valvidares, M.; Sessi, P.; Taylor, J. M.; Radu, F.; Chang, K.; Parkin, S. S. P. Intrinsic 2D-XY ferromagnetism in a van der Waals monolayer. *Science* **2021**, *374*, 616–620.

(4) Sasabe, N.; Ishii, Y.; Yamasaki, Y. X-ray magnetic circular dichroism originating from

the anisotropic magnetic dipole operator in collinear altermagnets under trigonal crystal field. *Phys. Rev. B* **2025**, *112*, 224401.
